# Zeta Potential and Size Analysis of Zeolitic Imidazolate Framework-8 Nanocrystals Prepared by Surfactant-Assisted Synthesis

**DOI:** 10.1021/acs.langmuir.3c03193

**Published:** 2024-03-15

**Authors:** Tristan K. Jongert, Ian A. Slowinski, Benjamin Dao, Victor H. Cortez, Thomas Gredig, Nestor D. Plascencia, Fangyuan Tian

**Affiliations:** † Department of Chemistry & Biochemistry, California State University Long Beach, Long Beach, California 90840, United States; ‡ Department of Physics & Astronomy, California State University Long Beach, Long Beach, California 90840, United States

## Abstract

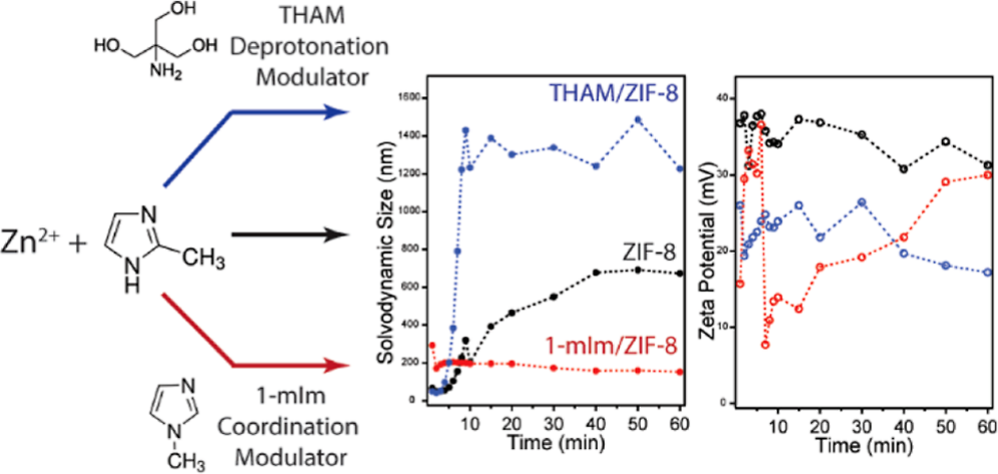

The crystal nucleation and growth mechanism of monodispersed metal–organic framework nanoparticles were studied using time-resolved light dynamic, electrokinetic, and powder X-ray diffraction methods. We confirmed that zeolitic imidazolate framework-8 (ZIF-8) nanocrystals follow a nonclassical crystal growth pathway, where a fast nucleation occurs with dense liquid clusters or nanocrystals forming spontaneously when two precursors are mixed. We also explored the zeta potential and solvodynamic size changes of ZIF-8 prepared by a surfactant-assisted synthesis. Three modulators, including 1-methylimidazole (1-mIm), tris(hydroxymethyl)aminomethane (THAM), and (1-hexadecyl)trimethylammonium bromide (CTAB), were studied. We found that 1-mIm dramatically increases the rate of nucleation of ZIF-8. With an increasing amount of 1-mIm, which functions as a coordination modulator, the size increases, and the zeta potential of ZIF-8 decreases. Whereas THAM, as both a coordination and a deprotonation modulator, increases the size and zeta potential of ZIF-8 simultaneously, CTAB, as a long alkyl cationic surfactant, mainly adsorbs on the surface of ZIF-8, and the zeta potential of the formed ZIF-8 is controlled by the amount of CTAB in solution compared with its critical micelle concentration. Overall, we reveal that the modulator type and concentration can be used to control the size and zeta potential of the dispersed ZIF-8 nanocrystals in a colloid system. The experiments also enable identification of the nucleation and crystal growth processes of ZIF-8. The findings will be applicable to other nanocrystals in colloid systems, which are used for heterogeneous catalysis and guest molecular loadings.

## Introduction

Zeolitic imidazolate framework-8 (ZIF-8) has been studied substantially as a model metal–organic framework (MOF) to understand MOF crystal growth in general. ZIF-8 is composed of tetrahedrally coordinated Zn^2+^ connecting with 2-methylimidazole (2-mIm) by forming a zeolitic sodalite topology. Due to its high Brunauer–Emmett–Teller specific surface area (up to 1960 m^2^/g),^1^ excellent solvo and thermal stability, ease of synthesis, and versatile postsynthetic properties, ZIF-8 has been studied for various applications, including gas capture and separation,^2^ water remediation,^3^ electrochemical sensing,^4^ and host–guest interactions in biological systems.^5^ ZIF-8 of Zn (II) was first reported by Chen’s group in 2006; these crystals were harvested after a month of reaction.^6^ Later that year, Yaghi’s group reported 12 ZIFs (ZIF-1–12); among all, ZIF-8 was synthesized by a solvothermal method involving dimethylformamide (DMF) at 140 °C for 24 h.^7^ In 2009, Cravillon et al. reported a rapid (60 min) room-temperature method of making ZIF-8 nanocrystals (∼45 nm).^8^ Since then, ZIF-8 has been widely used in many different studies due to its mild and simple synthesis conditions. In most cases, the specific particle size or morphology of the ZIF-8 crystals is needed for various applications. The formation process of ZIF-8 determines its final size and shape; therefore, it is critically important to understand how ZIF-8 is formed. So far, the formation of ZIF-8 has been confirmed with at least two stages: nucleation and crystal growth.^9−11^ A more recent mechanistic study led by Mirsaidov’s group revealed a liquid–liquid phase separation prior to the nucleation, adding a third stage of ZIF-8 formation.^12^ From the perspective of classical nucleation theory (CNT),^13−15^ a critical nucleus is formed in a synthesis solution; the size of the nucleus ranges from 10 molecules up to 40 nm.^16,17^ Due to a sharp boundary between the solution and critical nuclei, these nuclei are in a metastable phase and will further bond together to form a large nucleus, consequently becoming a crystal.^18^ Time-resolved in situ static light scattering studies indicated that ZIF-8 goes through a continuous nucleation process followed by a fast crystal growth.^11^ The same group also utilized in situ small-angle and wide-angle X-ray scattering to study the fast ZIF-8 nucleation; they observed prenucleation clusters formed at the early growth stage, suggesting that the crystallization of ZIF-8 may not follow the CNT.^10^ The other commonly accepted mechanism nowadays in solution crystallization is the nonclassical pathway. This describes immediate nucleation in a colloidal system, where in droplets of dense liquid (known as clusters) rapidly form and become crystal nuclei due to the ordered structure.^19,20^ The review by Karthika et al. explains the differences between CNT and the nonclassical pathway in a great depth.^18^ Transmittance electron microscopic (TEM) studies by Venna et al. confirmed that ZIF-8 went through a metastable phase through a semicrystalline-to-crystalline transformation.^21^ However, the metastable intermediates during ZIF-8 formation were also confirmed by in situ atomic force microscopic (AFM) studies.^22^ Patterson and co-workers used in situ liquid cell TEM and revealed that ZIF-8 forms nuclei within a few minutes and the crystal growth is a surface-limited process.^23^ However, there is still ambiguity in ZIF-8 crystal formation, especially in the presence of modulators. In this work, we use light dynamic and electrokinetic methods to study the nucleation and crystal growth of ZIF-8 nanoparticles in colloidal systems with and without modulators.

In the presence of a modulator (including additives, surfactants, and capping ligands), the size and shape of ZIF-8 can be tuned. We postulate that these changes result from different nucleation and growth pathways during the ZIF-8 crystal formation process. For example, nucleation rate has an important role in crystal size; a slow nucleation rate usually results in larger crystals and vice versa.^24^ Formate, 1-methylimidazole (1-mIm), and *n*-butylamine were studied on their effects on the size of ZIF-8.^11^ Both formate (p*K*_a_ = 3.86) and 1-mIm (p*K*_a_ = 7.06) have lower p*K*_a_ values than that of the ZIF-8 building ligand, 2-mIm, which prevents deprotonating the intermediate [Zn/2-mIm] clusters, causing a slower nucleation rate, thus generating larger ZIF-8 crystals. The p*K*_a_ value of 2-mIm is experimentally determined as 7.86 in water.^25^ We assume that the p*K*_a_ values measured in aqueous conditions are similar to the values for methanolic solutions based on a comparison of imidazole in both solvents.^26,27^*n*-Butylamine is a stronger base with a p*K*_a_ of 10.78, leading a faster deprotonation process, thus accelerating the nucleation rate and resulting in smaller ZIF-8 crystals.^11^ In another study, an opposite trend was observed when the concentration of 1-mIm was greater than 100 mM; the size of ZIF-8 crystals decreased with an increasing concentration of 1-mIm.^28^ Therefore, we re-examine the effect of 1-mIm on the ZIF-8 nucleation and crystal growth process in this work.

Surfactant-mediated synthesis can produce ZIF-8 with various particle sizes and shapes, which extends their applications in adsorption,^29^ drug delivery,^30^ and catalysis.^31^ Several groups have done extensive works on studying the effect of surfactants on ZIF-8 growth in terms of the crystallinity, particle and pore sizes, and morphology.^11,28,32−35^ These recent review articles have uncovered the fascinating findings in-depth.^36−38^ However, there is a lack of understanding of the surface charges of ZIF-8 nanocrystals dispersed in solvent with or without modulators, which is important to understand the crystal formation and their applications in colloids. Besides 1-mIm, we also chose tris(hydroxymethyl)aminomethane (THAM) and cetyltrimethylammonium bromide (CTAB) as the other two modulators. THAM has a slightly higher p*K*_a_ (8.07)^25^ than that of 2-mIm (7.86) and 1-mIm (7.06), and we reckon it will be a great comparison between THAM and 1-mIm on ZIF-8 formation since the p*K*_a_ of 2-mIm is in between these two compounds. CTAB is a cationic surfactant that has been used to study its effect on the shape of ZIF-8.^32,36,39^ Pan et al. reported that CTAB preferentially adsorbs on the [100] facets of ZIF-8 in aqueous synthesis conditions.^33^ On a follow-up work, Pan’s lab generated a morphological map of ZIF-8 based on CTAB concentrations in aqueous conditions.^34^ However, the effect of CTAB on ZIF-8 morphological change has not yet been studied in alcohol systems yet. This work will examine the size and zeta potential (ζ-potential) effects of these modulators on ZIF-8 that is synthesized in alcohol. Despite its intrinsic porous structure, ZIF-8 has an extremely low uptake amount of ethanol. Therefore, we used ethanol as the dispersion system for our ζ-potential studies. ζ-potential plays an important role in colloid stability, flotation, adsorption, and coagulation/flocculation.^40^ Surfactants can induce flotation by changing the wettability of the solids. To our knowledge, most of the ζ-potential works reported so far for ZIF-8 were only using ζ-potential as a tool to confirm its stability in colloids; no work has been done to systematically study the ζ-potential correlating to ZIF-8 nucleation and crystal growth, not to mention the formation of ZIF-8 in the presence of modulators.

## Experimental Section

### Chemicals and Materials

All chemicals were reagent grade or better, used as-received, and included zinc nitrate hexahydrate (Zn(NO_3_)_2_·6H_2_O, Fisher Chemical, ACS Certified), 2-mIm (Acros, 99%), 1-mIm (Thermo Scientific, 99%), CTAB (Alfa Aesar, 98%), THAM (Fisher Chemical, ACS Certified), methanol (Fisher Chemical, ACS grade), and ethanol (Thermo Scientific, 200 Proof).

### ZIF-8 Synthesis by the Surfactant-Assisted Method

ZIF-8 nanocrystals were synthesized using the rapid room-temperature method according to the literature with modifications.^3^ The synthesis process was varied through the introduction of different modulators. The benchmark synthesis method was used to compare the role of the modulator in the process. A molar ratio of Zn(NO_3_)_2_·6H_2_O to 2-mIm to methanol of 1:8:988 was used. Zn(NO_3_)_2_·6H_2_O (0.7333 g) and 2-mIm (1.622 g) were dissolved in separate portions of methanol (50 mL). They were combined and stirred rapidly for an hour. The ZIF-8 nanocrystals were separated from methanol by centrifugation at 10,000 rpm for 15 min. The resulting crystals were rinsed, dispersed into ethanol, and then centrifuged twice over. Finally, the ZIF-8 nanocrystals were redispersed into 25 mL of ethanol. Three surfactants, 1-mIm, THAM, and CTAB, were introduced to the 2-mIm solution, respectively, prior to being mixed with the Zn^2+^ solution. Following a similar procedure, the combined Zn^2+^ and 2-mIm together with the surfactant was stirred for an hour to make individual surfactant-assisted ZIF-8. Based on a previously reported method using 1-mIm at various concentrations for ZIF-8 size control,^41^ we added various amounts of 1-mIm (397, 626, 803, 1006 μL) to the 2-mIm solution, corresponding to a molar ratio of 2-mIm:1-mIm at 1:*X* (*X* = 0.24, 0.37, 0.48, 0.60). We used CTAB concentrations at 0.3, 0.7, 1.17, and 2.74 mM based on its critical micelle concentration (CMC) in methanol which is at 1.0 mM.^42^ We evaluated the potential effects of CTAB micelles on ZIF-8 synthesis. Zheng et al. reported that 10 mM THAM has a morphological influence on ZIF-8.^32^ In this study, THAM was weighed out as 25, 50, 100, and 175 mg, corresponding to 2.06 4.12, 8.25, and 14.4 mM, to synthesize THAM-assisted ZIF-8. The resulting ZIF-8 nanocrystals were characterized using the following techniques.

### Characterization

#### Dynamic Light Scattering

The solvodynamic size and ζ-potential of the ZIF-8 nanocrystals were measured on a Malvern Zetasizer Nano ZS90. Both sets of measurements were performed at 25 °C using a material reflective index of 1.380 for ZIF-8. Methanol was used as the dispersed phase for kinetics analysis with a reflective index of 1.326 and a viscosity of 0.5476 cP. For nonkinetics measurements, we used ethanol as the dispersant with a reflective index of 1.386 and a viscosity of 1.10 cP. Three runs were collected per measurement with positioning, conductivity, and duration automatically set. The solvodynamic size measurements used dynamic light scattering (DLS) in disposable clear square cuvettes with Mark–Houwink parameters, A-parameter 0.428 and K-parameter 7.67 × 10^–5^, and a 90° detector. ζ-potential measurements utilized electrophoretic light scattering (ELS) with a Smoluchowski model *F*(κa) value of 1.50. ZIF-8 is considered as solid, near-spherical, nonconducting particles in this study when we fit the zeta potential using the Smoluchowski model. For kinetic analysis, we monitored zeta potential and size changes of ZIF-8 during its synthesis process. We collected a sample of 875 μL of ZIF-8 solution every minute for the first 10 min and then at 15, 20, 30, 40, 50, and 60 min until the synthesis was completed after an hour. The collected solution was analyzed for zeta potential and size right away using a dip cell and disposable cuvettes. The data was processed with a mean of each run for a faster scan. Ethanol-dispersed solution ζ-potentials were measured in disposable folded capillary cells.

#### Powder X-ray Diffractometer

Powder X-ray diffraction (PXRD) data was collected using a Bruker D2 Phaser with a Cu Kα source (λ = 1.5406 Å) at 30 kV. The diffraction angle of measurements was between 5 and 40° with a step size of 0.02°. The ZIF-8 samples in ethanol were prepared by placing drops of ZIF-8 solution onto a silicon low background holder and left to air-dry until only a thin layer of powder remained. For time-resolved PXRD studies, ZIF-8 samples were prepared by taking a constant volume (2 mL for the first 10 min and 1 mL for later time) of ZIF-8 synthesis methanol solution at every minute for the first 10 min and then at 20, 30, and 60 min. The solution was centrifuged at 10,000 rpm in microcentrifuge tubes for 1 min. Then, the collected powder was dispersed back to a minimum amount of methanol in the same container. Finally, the colloidal solution was transferred drop-by-drop to a Bruker silicon low background holder and was left to air-dry before PXRD analysis.

#### Attenuated Total Reflectance Infrared Spectroscopy

Attenuated total reflectance infrared spectroscopy (ATR-IR) spectra were obtained using a Bruker Alpha I Platinum infrared spectrometer. The spectra were scanned in the range of 4000–400 cm^–1^ with a resolution of 4 cm^–1^ and 16 scans per spectrum. Prior to measurements, the samples were dried overnight in a vacuum oven at 70 °C to remove the residual solvent. Ambient air was used as the background.

#### Atomic Force Microscopy

AFM images were collected using a Park XE7 instrument. We used multipurpose PPP-NHCR cantilevers with a resonance frequency around 350 kHz. The topography images were measured in noncontact mode. All AFM samples were prepared by adding a drop of ZIF-8 synthesis solution to a precleaned Si (111) chip (approximately 1 × 1 cm^2^) and then left to air-dry. Silicon chips were cleaned by sonicating in methanol for 5 min and then dried with N_2_ gas prior to use.

## Results and Discussion

### Size and ζ-Potential Measurements of Pristine ZIF-8

**Figure 1:**
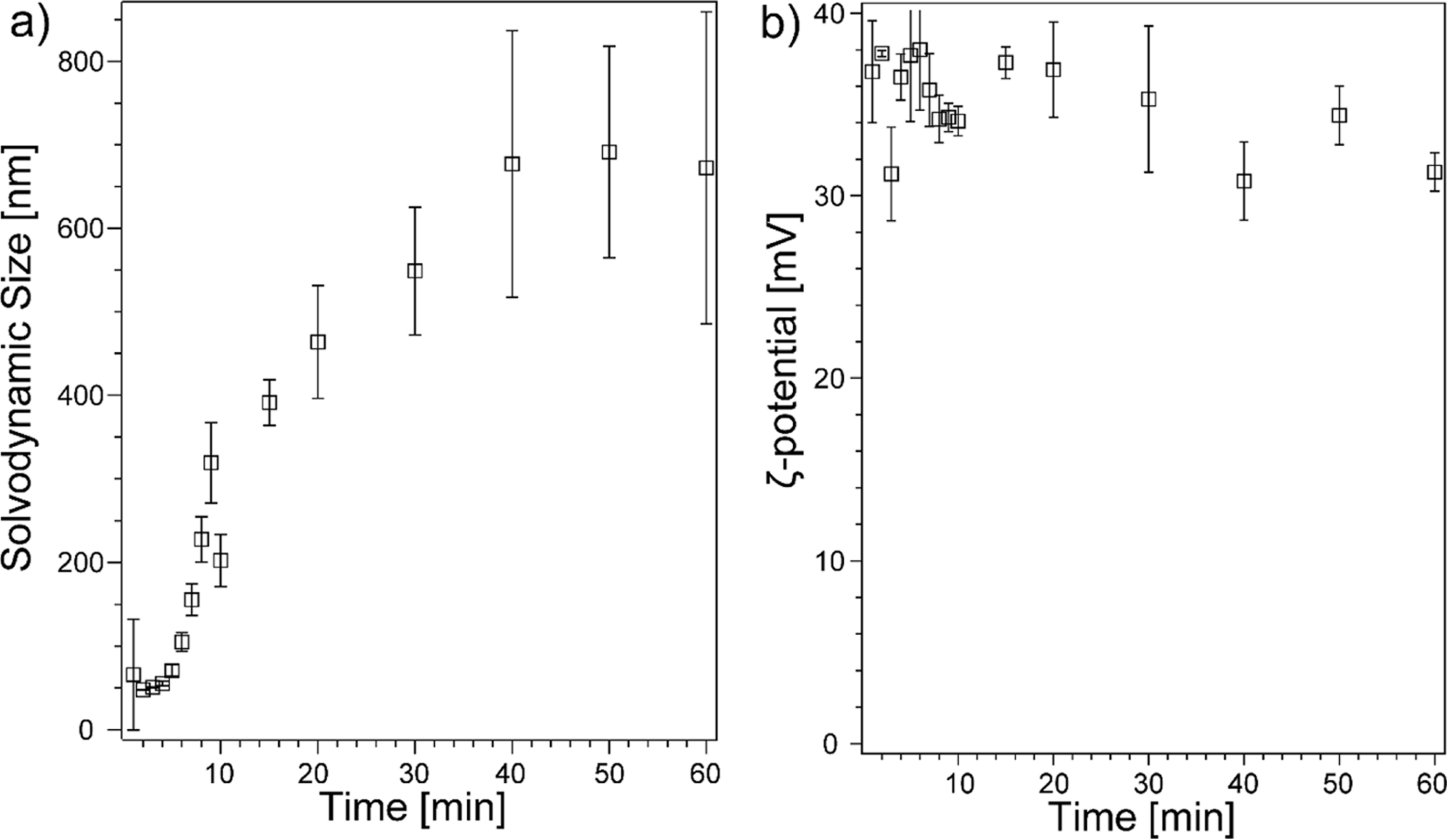
Solvodynamic size (a) and ζ-potential (b) of ZIF-8 in methanol as a function of time during the synthesis. Error bars indicate the standard deviations from at least three trials.

**Figure 2:**
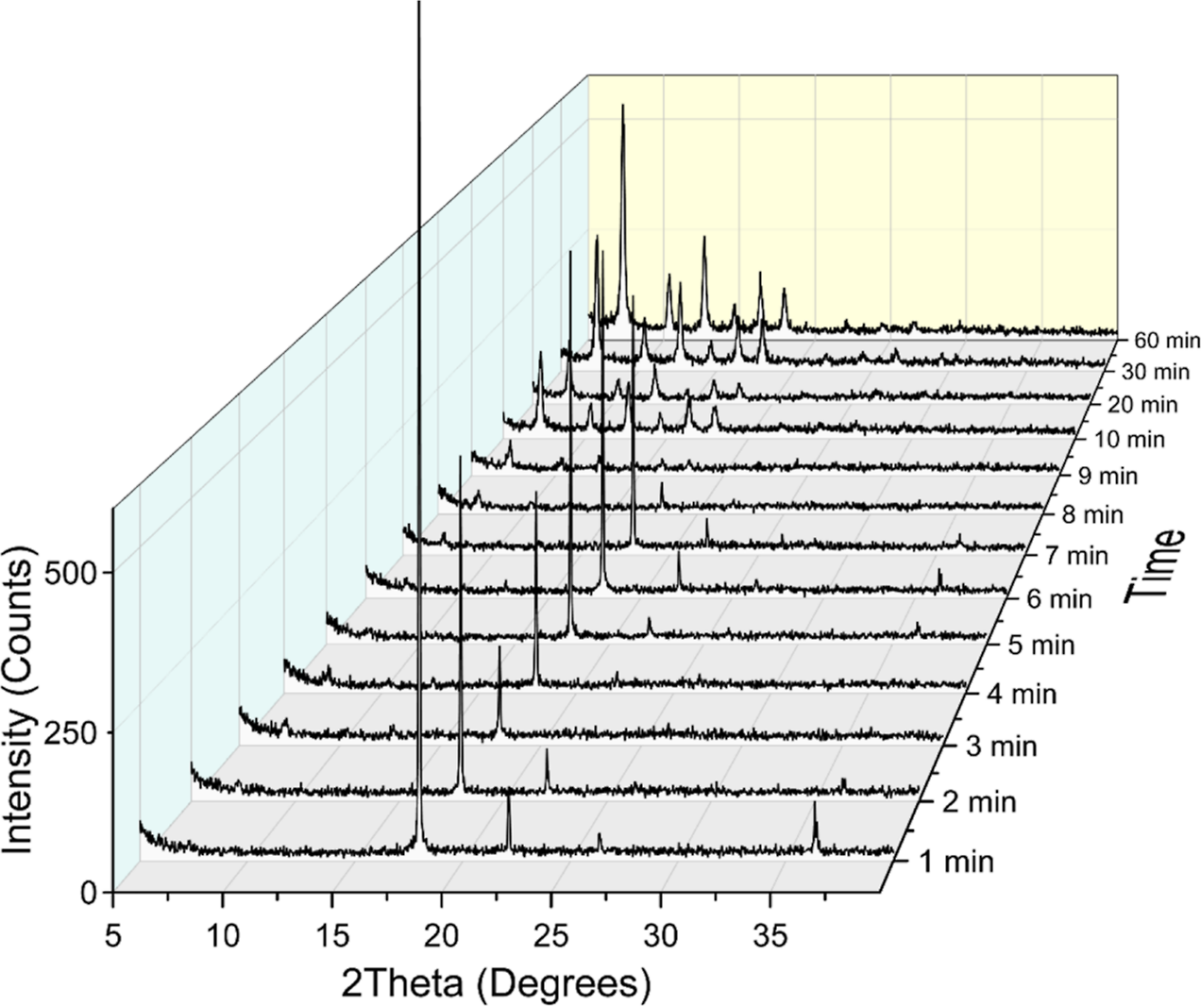
Time-resolved PXRD patterns of pristine ZIF-8 over the synthesis time.

DLS and ELS analyses were performed to monitor the solvodynamic size and ζ-potential of ZIF-8, respectively, as a function of time during ZIF-8 synthesis with and without modulators. We first studied the nucleation and crystal growth process of pristine ZIF-8 with time-resolved DLS and ELS kinetics along with PXRD studies. Figure 1a shows the plot of the solvodynamic size of ZIF-8 during its synthesis in methanol. An average solvodynamic size of 55.1 ± 9.8 nm of ZIF-8 was formed within the first 3 min, indicating that nanocrystals/clusters form spontaneously after mixing the Zn^2+^ and 2-mIm solutions. The crystal solvodynamic size increases dramatically in the first 10 min; then, the growth slows down and shows a linear trend between 10 and 40 min. Eventually, the solvodynamic size of ZIF-8 reached a plateau at around 680 nm after 40 min. The increase of the solvodynamic size is an indicator of crystal growth; the fast crystallization of ZIF-8 slows down after 10 min, which is consistent with previously reported XRD phase transformation studies that ZIF-8 can reach a metastable phase after 20 min of mixing.^21,43^ To validate the size measured in DLS, we collected the AFM images of ZIF-8 after 10 min mixing (Figure S1). The particle size measured by AFM was found to be ∼103 nm, which is smaller than that of our DLS result. The solvodynamic size of the formed ZIF-8 is significantly larger than the size reported using SEM or TEM (55–120 nm),^10,44,45^ which is possibly due to the solvation shell formed around ZIF-8 and the strong hydrogen bonding that may link solvated ZIF-8 together. Figure 1b shows the plot of the ζ-potential of ZIF-8 colloidal solution versus time during synthesis. Measured ζ-potential values stay in the range of 30.8 and 38.0 mV throughout the mixing process. Despite ζ-potential being a poor quantitative indicator of surface charge, the sign of ζ-potential reveals the nature of surface charge (positive or negative) of colloid particles. Based on our ELS results, we confirmed that ZIF-8 is positively charged in the colloidal solution. This result is consistent with the result of our previous X-ray photoelectron spectroscopic studies that nanoscale ZIF-8 has a Zn-rich surface,^44^ which will result in predominant Zn^2+^ on the electric double layer (EDL) of ZIF-8 in a colloid system. ζ-potential jumps up to ∼37 mV at the first 2 min during the reaction, again suggesting a spontaneous formation of ZIF-8 nanocrystals or dense liquid ZIF-8 clusters upon mixing 2-mIm and Zn^2+^ solutions. Although different crystallization speeds are revealed by the solvodynamic size changes, the ζ-potential exhibits little variation, indicating that the surface charge of ZIF-8 nanocrystals/clusters remains stable regardless of solvodynamic size. To further understand the crystallinity changes of ZIF-8 during its formation, we compared the powder X-ray diffraction (PXRD) patterns of ZIF-8 as a function of the synthesis time. The PXRD data of pristine ZIF-8 is summarized in Figure 2. The characteristic [011] diffraction peak of ZIF-8 at 2θ = 7.3° started showing up at 3 min, indicating a fast formation of ZIF-8; this is consistent with our electrokinetic results. The peaks at 17.9, 26.4, and 36.3° shown at 1 min are associated with 2-mIm, confirmed by the PXRD pattern of 2-mIm, as shown in Figure S4. The peaks associated with 2-mIm disappeared after 7–8 min. The peak around 18° became more obvious after 10 min, which is associated with the ZIF-8 [222] diffraction peak. At 10 min and after, all the ZIF-8 peaks were visible, indicating the crystal growth of ZIF-8. By combining our DLS, ELS, and PXRD results, we confirmed that ZIF-8 follows a nonclassical crystal growth pathway. When two precursors are mixed, a rapid nucleation occurs, forming dense liquid clusters or nanocrystals, as shown by a high ζ-potential. Additionally, ZIF-8 shows a two-phase crystal growth (with fast and slow rate constants) within the first 40 min, which is consistent with previously reported nanocrystal growth in colloids.^46^

### Size and ζ-Potential Measurements of ZIF-8 with Modulators

**Figure 3:**
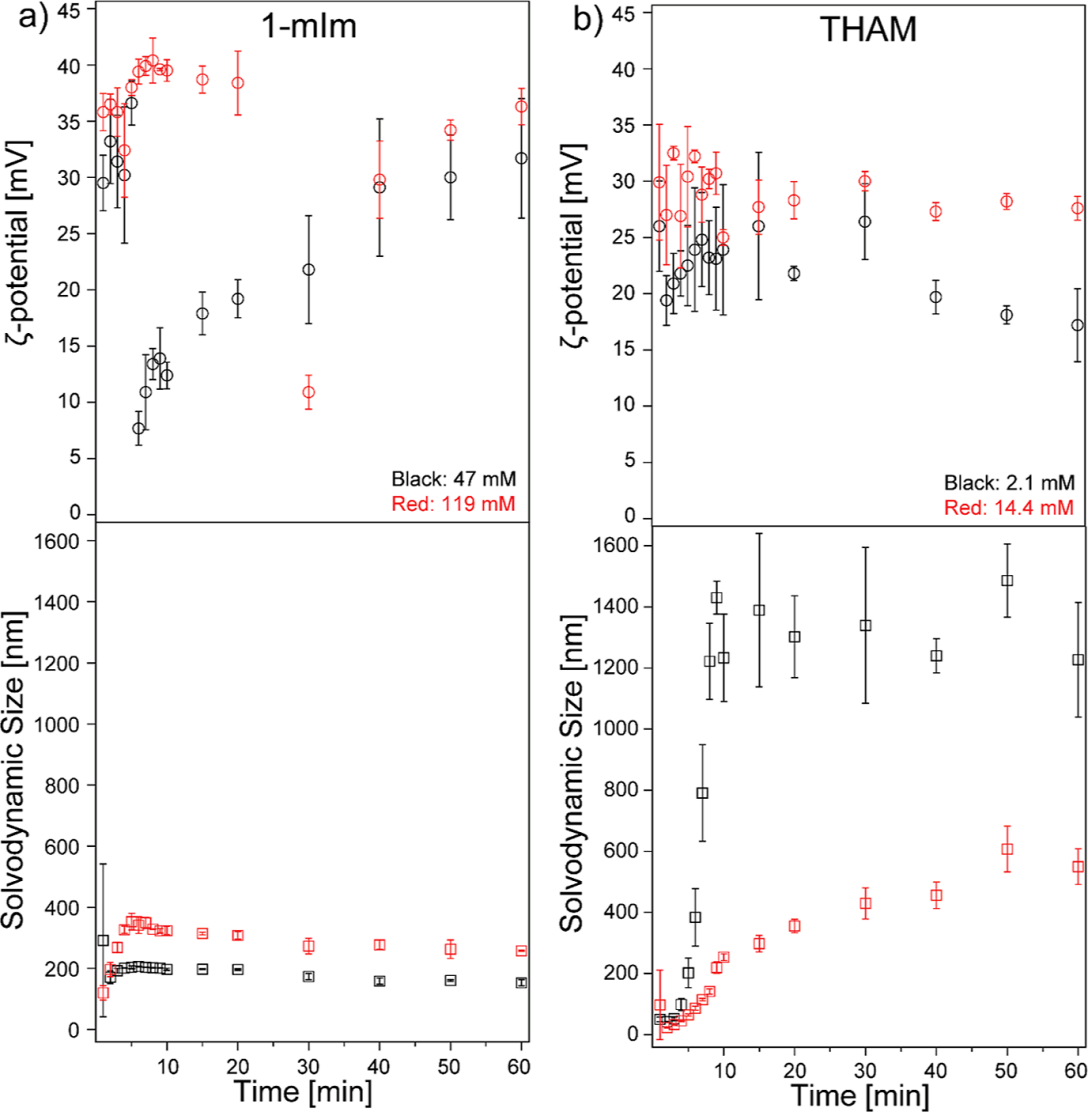
ζ-potential (top) and solvodynamic size (bottom) of ZIF-8 in methanol synthesized with the addition of 1-mIm (a) and THAM (b) at two different concentrations in methanol as a function of synthesis time. Error bars indicate the standard deviations from at least three trials.

Modulators can influence MOF crystal nucleation or crystallization, thus altering the crystal size and morphology. According to Jiang et al.,^47^ MOF modulators can be divided into two categories: coordination modulators and deprotonation modulators. Coordination modulators are the molecules that compete with ligands and break coordination balance during crystal growth.^48,49^ Deprotonation modulators can facilitate the deprotonation of ligands due to their high p*K*_a_ values.^47^ During the ZIF-8 synthesis, both 1-mIm and THAM can function as coordination modulators to compete with 2-mIm. Since THAM has a higher p*K*_a_ than that of 2-mIm, it can also play a role as a deprotonation modulator. 1-mIm has been studied for its effect on ZIF-8 crystal size and morphology.^11,28,36,50^ Herein, we chose two extreme concentrations (high and low at 119 and 47 mM, respectively) of 1-mIm and studied its effect on the size and ζ potential during ZIF-8 formation. As shown in the bottom of Figure 3a, when 1-mIm was added into the ZIF-8 synthesis solution, the solvodynamic size exhibited a similar trend as that observed in pristine ZIF-8. However, we noticed a sharper slope on the solvodynamic size growth, indicating a much faster nucleation rate within the first 5 min. The solvodynamic size reached a plateau after 5 min for both high and low concentrations of 1-mIm-assisted ZIF-8. Also, we noticed that the solvodynamic size dropped to 152 and 257 nm for low and high concentrations of 1-mIm-assisted ZIF-8, respectively, which are much smaller compared to those of pristine ZIF-8 (∼680 nm). We believe that this is due to 1-mIm as a coordination modulator competing with 2-mIm and participating in the coordination bonding of Zn clusters, promoting a faster nucleation rate, thus resulting in smaller crystal sizes. As indicated by our density functional theory (DFT) simulations, Zn^2+^ can bind to the imidazole nitrogen in both 1-mIm and 2-mIm (Figures S5 and S6). 1-mIm accelerates the nucleation of ZIF-8 clusters, which is confirmed by comparing the slopes of size growth shown in Figures 1a and 3a for pristine ZIF-8 and 1-mIm-assisted ZIF-8, respectively. The quickest size growth for pristine ZIF-8 is noticed between 5 and 10 min, while the fastest growth for 1-mIm-assisted ZIF-8 is within the first 5 min. Faster nucleation typically leads to smaller crystal sizes (nanoscale).^51^ Once Zn^2+^ bonded to 1-mIm, the next linking process is disrupted since 1-mIm cannot go through further deprotonation, thus no neighboring Zn^2+^ can be bonded to the formed [Zn/1-mIm] complexes. The amount of 1-mIm added to ZIF-8 synthesis solutions influences the solvodynamic size of ZIF-8 during the crystal growth process; a higher concentration of 1-mIm (119 mM) resulted in a slightly larger solvodynamic size, compared to the case with a lower concentration (47 mM). However, the ζ-potential trends are remarkably different in these two cases. Figure 3a shows that the ζ-potential increases to ∼35 mV within the first 5 min when 47 mM 1-mIm was mixed with the ZIF-8 synthesis solution, and it drops to 7 mV after 5 min and slowly climbs back to 30 mV over the next 55 min of stirring, following an almost linear trend. However, the ζ-potential of ZIF-8 solution mixed with 119 mM of 1-mIm remains in the range of 30–40 mV with one exception at 11 mV observed at 30 min. The sudden ζ-potential decrease observed in the low 1-mIm concentration condition can be related to the disruption of surface charge that is not related to size growth.^52^

When THAM was used as a modulator, we noticed that the ζ-potential showed a trend similar to that of the pristine ZIF-8, and there was no statistical difference between the two concentrations of THAM added, as shown in Figure 3b top. However, we observed a substantial difference in solvodynamic size corresponding to the concentration of THAM added to the ZIF-8 synthesis solution, as shown in Figure 3b bottom. With a higher THAM concentration (14.4 mM), the solvodynamic size of ZIF-8 stabilizes at ∼600 nm after 60 min; the size is dramatically smaller than the ones prepared with a lower concentration (2.1 mM), which resulted in a solvodynamic size around 1300 nm. The reason for the difference can be associated with the interactions between THAM and Zn^2+^ during the nucleation process. As suggested by our DFT studies (Figure S7), THAM can form coordination bonds between deprotonated hydroxyl groups and open metal sites in the Zn clusters during ZIF-8 formation. Besides, free Zn^2+^ ions can chelate between a hydroxyl group and a deprotonated amine group within a THAM molecule by forming a five-membered ring. If Zn^2+^ links between two hydroxyl groups within a THAM molecule, then a six-membered ring can be formed. In either case, ZIF-8 nucleation process will be disrupted by the presence of THAM. Also, THAM has a slightly higher p*K*_a_ value (8.07) than that of 2-mIm (7.86); however, the value is not as high as the p*K*_a_ of the Zn^II^-coordinated imidazole complex, which is around 10.3.^11,53^ This indicates that THAM can promote the deprotonation of 2-mIm, but it may not deprotonate the [Zn/2-mIm] clusters during ZIF-8 formation. A higher concentration of THAM, as a coordination modulator, enables the interruption of more nucleation sites, thus causing smaller solvodynamic sizes. In other words, THAM functions as both coordination and deprotonation modulators; when its concentration is high in the ZIF-8 synthesis solution, it mainly functions as a coordination modulator. However, when a lower concentration of THAM is used, only a limited amount of initial ZIF-8 nuclei are affected, and the majority of Zn^2+^ are still tetrahedrally coordinated with 2-mIm. In this case, THAM can function as a bridging agent in the solvent to link ZIF-8 nanocrystals together to form aggregates, as observed in our previous AFM work.^54^

**Figure 4:**
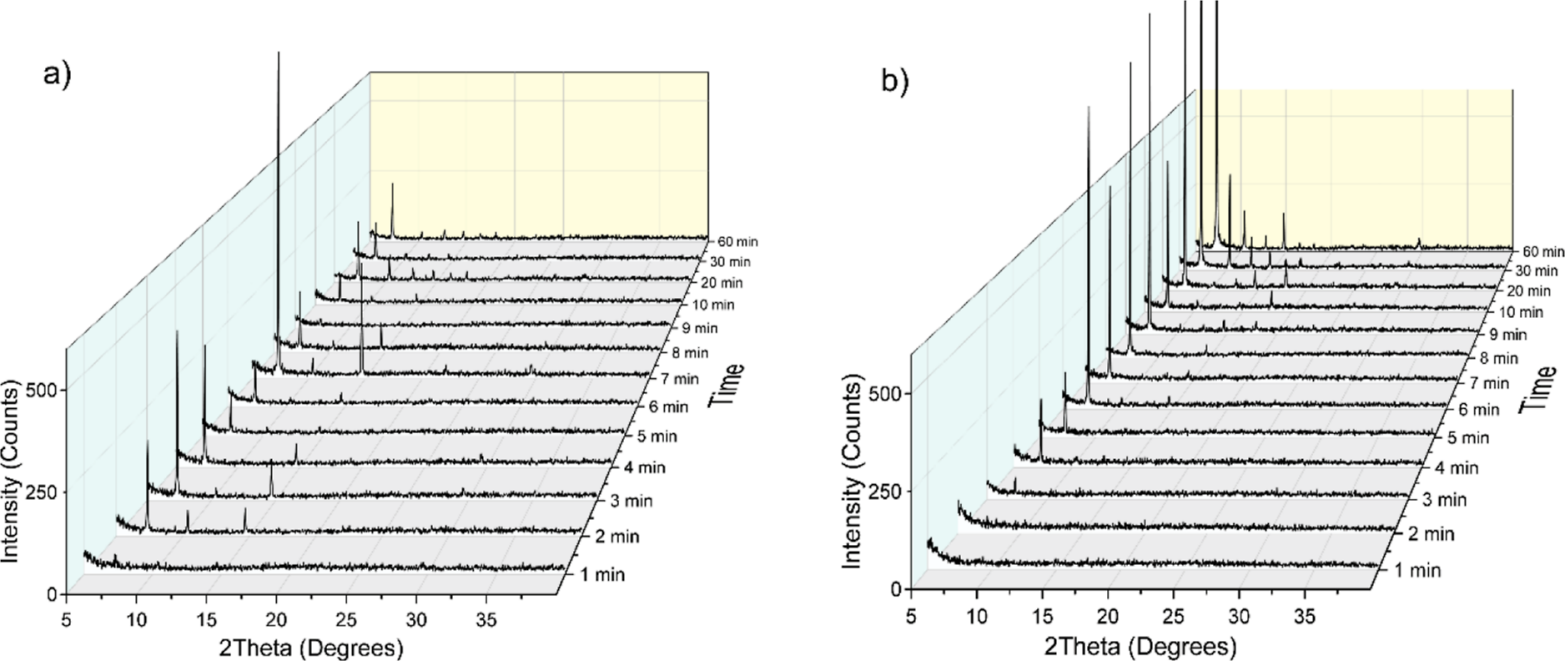
Time-resolved PXRD patterns of ZIF-8 synthesized with additional (a) 119 mM of 1-mIm and (b) 2.1 mM of THAM over 60 min of synthesis time.

We further compared the crystallinity changes of ZIF-8 synthesized with 1-mIm and THAM during the synthesis processes. Figure 4 exhibits the PXRD patterns of ZIF-8 synthesized with 119 mM 1-mIm (1-mIm:2-mIm = 0.6:1) and 2.1 mM THAM (THAM:2-mIm = 0.3:1, molar ratios in both cases). The reasons for choosing these two concentrations were because (1) the condition with the highest concentration of 1-mIm is a good representative of the competition between 2-mIm and 1-mIm, which behaves as a coordination modulator; and (2) the condition with the lowest concentration of THAM shows unique solvodynamic sizes of ZIF-8 in our DLS studies (Figure 3b). We noticed that the 1-mIm-assisted ZIF-8 exhibited the characteristic ZIF-8 [011] diffraction peak at 7.3° starting at 1 min (Figure 4a), and several other ZIF-8 planes were visible at 2 min, suggesting a faster nucleation process compared to that of pristine ZIF-8, which is consistent with our electrokinetic results. As for THAM-assisted ZIF-8 (Figure 4b), the [011] diffraction peak started showing up after 3 min, indicating a nucleation rate similar to that of the pristine ZIF-8. However, comparing with the PXRD patterns of ZIF-8 shown in Figure 2, THAM-assisted ZIF-8 shows clearer and sharper ZIF-8 features, meaning a faster crystal growth over time with THAM in the presence in the ZIF-8 synthesis solution. We think the small amount of THAM mainly functions as a deprotonation modulator, which promotes deprotonating 2-mIm, thus facilitating further crystal growth. In both 1-mIm- and THAM-assisted ZIF-8 conditions, we did not observe 2-mIm peaks at earlier reaction time. We are not sure about the reasons, but we think it may be related to the interactions between 2-mIm and surfactants in the solvent.

**Figure 5:**
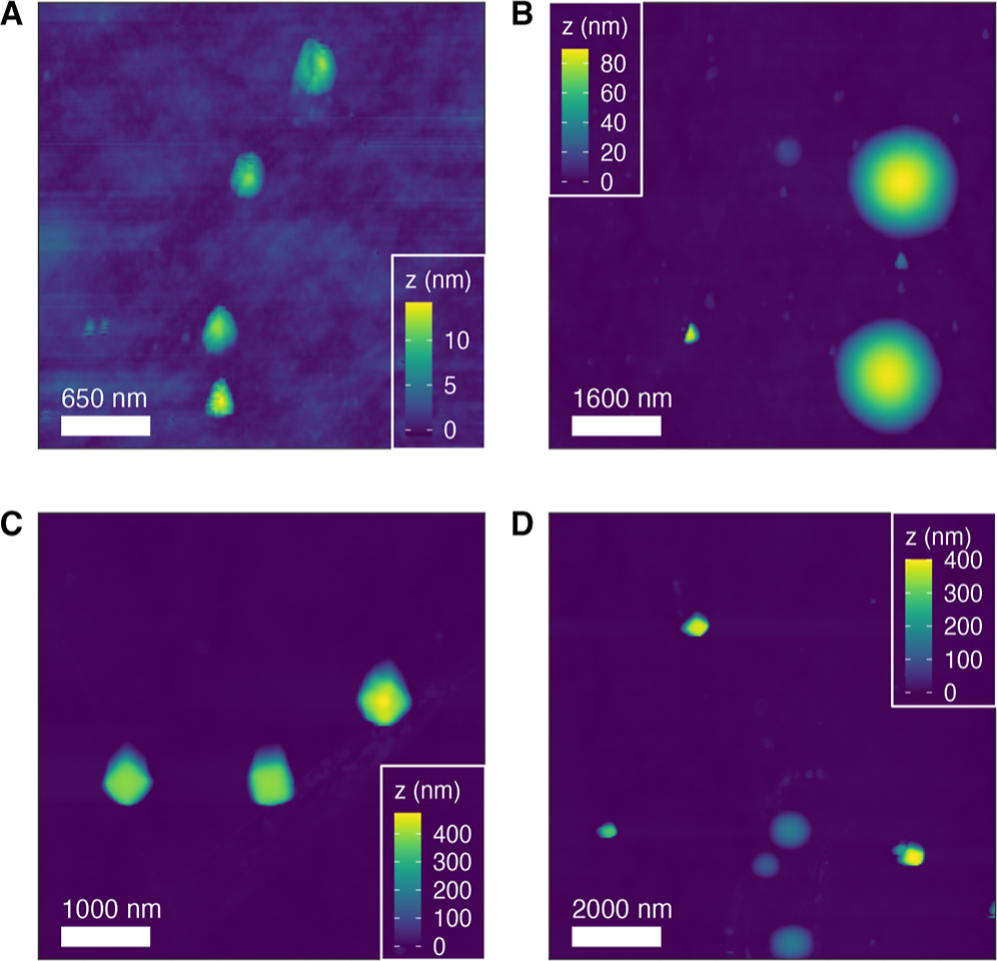
AFM topography images of ZIF-8 synthesized with an additional 119 mM of 1-mIm at 2 min (A), 10 min (B), 20 min (C), and 60 min (D).

Additionally, we performed AFM studies on 1-mIm- and THAM-assisted ZIF-8, especially to monitor particle formation at an early stage. Based on our electrokinetic and PXRD results, adding 1-mIm can promote ZIF-8 nucleation, as confirmed by all ZIF-8 features being visible on XRD at 2 min of synthesis time. We took AFM images of 1-mIm-assisted ZIF-8 after 2, 10, 20, and 60 min in the synthesis solution with additional 119 mM of 1-mIm, as shown in Figure 5. The cross-sectional line profiles to identify particle size are shown in Figure S3. We observed the crystals grow over time with a particle height (*z* value) from ∼10 at 2 min–90 nm at 10 min, 440 at 20 min, and 380 at 60 min. These values aligned with our DLS results (Figure 3a). It confirms that the growth of 1-mIm-assisted ZIF-8 crystals occurs from 2 to 20 min, and the particle has been fully grown at a time of 20 min with the additional 1-mIm. We also examined the THAM-assisted ZIF-8 using AFM. Based on our PXRD studies, the nucleation rates were found to be similar for pristine ZIF-8 and THAM-assisted ZIF-8; we looked at both samples after 10 min synthesis time under AFM (Figures S1 and S2). The particles synthesized with additional THAM were not as uniform as pristine ZIF-8 particles (Figure S1). All THAM-assisted ZIF-8 particles exhibited a height of around 56 nm with irregular shapes. The AFM-measured particle size is dramatically smaller than the value measured in our DLS analysis. We think the large solvodynamic size measured by DLS may be related to the strong surface interactions among ZIF-8 particles in the presence of THAM in methanol, which may form stable ZIF-8 aggregates through hydrogen bonding, as we discussed above.

### Modulator Effect on the Size and ζ-Potential of ZIF-8

**Figure 6:**
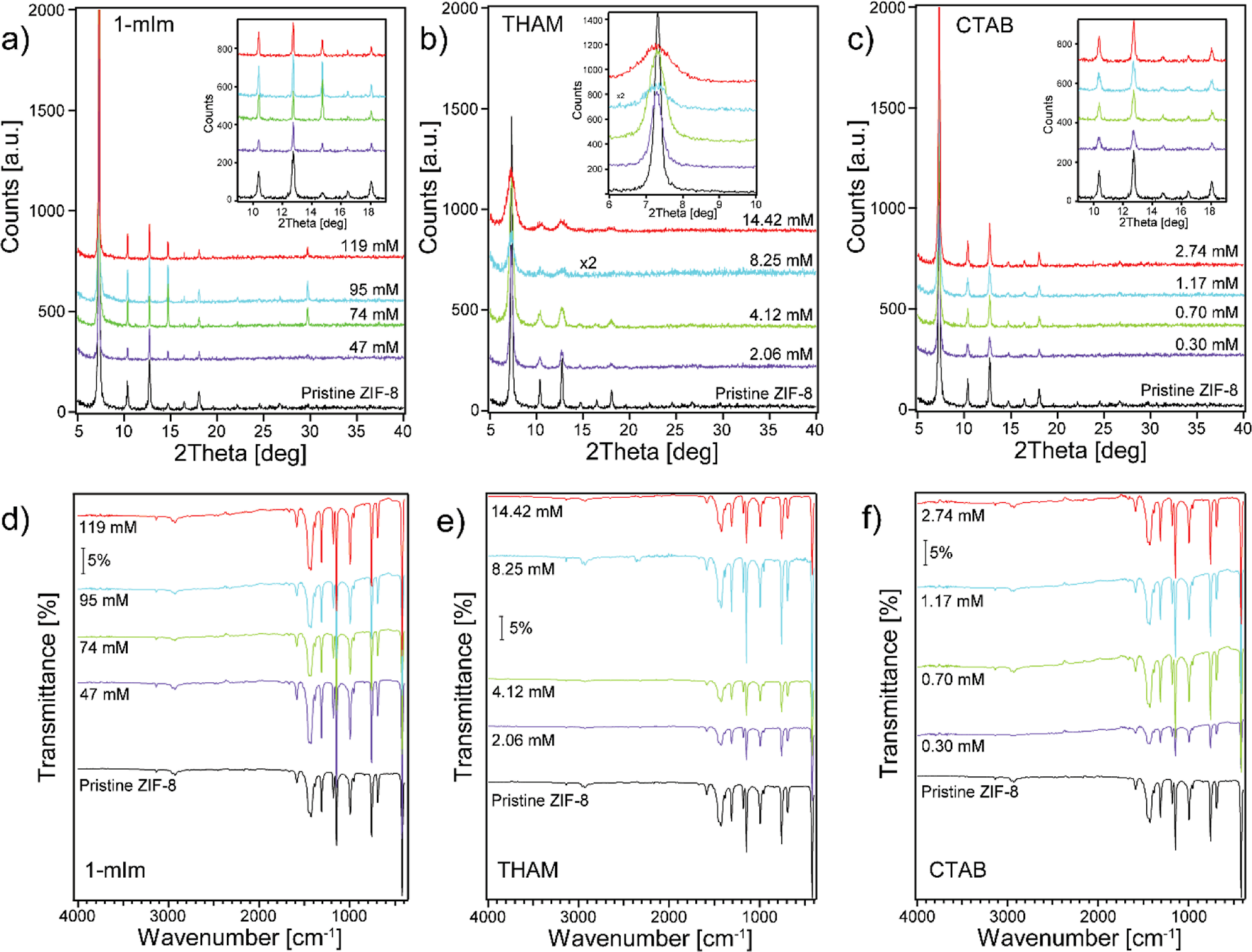
PXRD patterns (a–c) and ATR-IR spectra (d–f) of ZIF-8 synthesized with various amounts of 1-mIm, THAM, and CTAB.

Once ZIF-8 was synthesized in methanol, we redispersed it into ethanol, which resulted in a more stable colloidal solution with a longer lifetime. We first confirmed the crystal structures and chemical compositions of the modulator-synthesized ZIF-8 by comparing them with the results collected on pristine ZIF-8, as shown in Figure 6. The majority of ZIF-8 prepared by surfactant-assisted synthesis is identical to pristine ZIF-8 with a few exceptions. We noticed that the peak at 2θ of 29.7° increases with a higher 1-mIm concentration (Figure 6a). This peak is associated with the [044] plane of ZIF-8, as confirmed by our simulation results (Table S1). We believe the enhancement of this peak is related to the shape change of ZIF-8 with an increasing amount of the 1-mIm modulator, as we observed in our AFM studies (Figure 5C). Previous literature reported that the shape of ZIF-8 can evolve from truncated rhombic dodecahedra to rhombic dodecahedra with the addition of 1-mIm.^28,36^ With an increasing amount of THAM, the PXRD peaks of ZIF-8 were broadened (Figure 6b), indicating that the crystallinity of ZIF-8 synthesized with THAM was dampened. This may be due to the higher amount of THAM disrupting the coordination between Zn^2+^ and 2-mIm. Moreover, the additional THAM may promote the chelation between Zn^2+^ and THAM, thus further disturbing the crystal formation of ZIF-8. CTAB as a cationic surfactant has been confirmed to not participate in Zn coordination in our simulation work (Figure S8). Previous literature showed that CTAB prefers adsorbing on the [100] facets of ZIF-8.^33^ We noticed little effect of the CTAB altering crystal structure or chemical composition of ZIF-8, as indicated in Figure 6c,f.

We also compared the solvodynamic size and ζ potential of ZIF-8 in ethanol as a function of the modulator concentrations. The results are summarized in Table 1. To avoid the influence of nanoparticle concentration,^55^ we kept the concentrations of ZIF-8 nanocrystals in all measured solutions the same for particle size and ζ-potential measurements. Polydispersity index (PDI) values smaller than 0.2 were observed for pristine ZIF-8, all 1-mIm-assisted, and most THAM-assisted ZIF-8, indicating that monodispersed ZIF-8 colloid systems were formed in these cases. CTAB-assisted ZIF-8 exhibited greater PDI values (>0.2), suggesting a broader solvodynamic size distribution of these ZIF-8 nanoparticles. Additionally, we noticed that the size of 1-mIm-assisted ZIF-8 increases with an increasing amount of 1-mIm, which is consistent with the findings reported by Cravillon et al.^11^ However, the ζ-potentials of these 1-mIm assisted ZIF-8 are slightly greater than the value measured on pristine ZIF-8 (32.5 ± 0.6 mV). ζ-potential is often used to predict colloid stability. Based on the Derjaguin, Landau, Verwey, and Overbeek (DLVO) theory, colloid stability is in fact determined by the sum of van der Waals attractive forces and electrostatic repulsive forces.^40^ ζ-potential only reflects the repulsive interactions among colloidal particles as it is measured by tracking particle mobility in an electric field. Mobility depends on the nature of EDL, which can be affected by pH, ionic strength, and concentration.^52^ Therefore, the ζ-potential is a good indicator of colloid stability within the same system, but it is not enough to compare the stability among different colloidal systems. There are examples of highly stable systems with low ζ-potential, such as colloidal silica.^56,57^ Therefore, in this work, we only compare the ζ potential within the same colloid system but not in-between.

**Table 1:** Summary of Solvodynamic Size and ζ-potential of ZIF-8 Nanocrystals in Ethanol

ligand and concentration (mM)	particle size (d nm)	PDI	zeta potential (mV)
pristine ZIF-8	157.6 ± 0.5	0.145	32.5 ± 0.6
1-mIm			
47	51.1 ± 0.4	0.051	39.0 ± 2.0
74	115.1 ± 0.6	0.243	43.9 ± 1.9
95	170.1 ± 2.9	0.129	42.4 ± 0.9
119	268.5 ± 3.0	0.036	40.3 ± 1.0
CTAB			
0.3	145.3 ± 1.2	0.229	29.1 ± 0.7
0.7	163.8 ± 0.8	0.243	32.6 ± 0.3
1.17	164.2 ± 1.3	0.244	32.4 ± 0.6
2.74	145.9 ± 1.0	0.310	31.1 ± 0.4
THAM			
2.06	187.7 ± 4.5	0.121	23.0 ± 0.8
4.12	163.8 ± 1.7	0.159	24.3 ± 0.2
8.25	268.8 ± 0.4	0.273	27.8 ± 0.8
14.4	285.2 ± 3.5	0.035	38.5 ± 0.7

**Figure 7:**
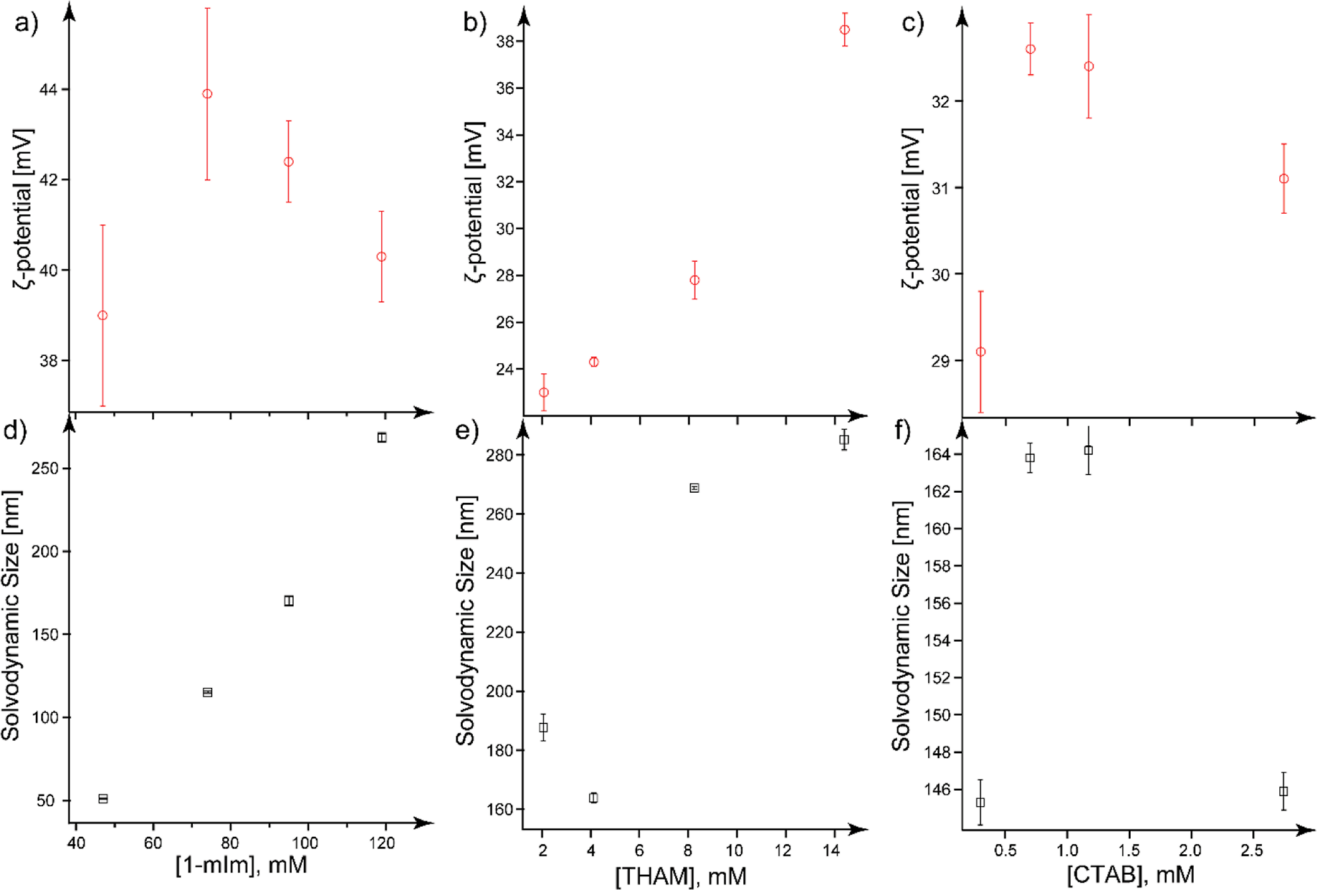
Trends of ζ-potential (a–c) and solvodynamic size (d–f) of ZIF-8 in ethanol as a function of the concentrations of 1-mIm (a,d), THAM (b,e), and CTAB (c,f). Error bars indicate the standard deviations from at least three trials.

Figure 7 compares the solvodynamic size and ζ-potential of ZIF-8 dispersed in ethanol as a function of the concentration of modulators, including 1-mIm, THAM, and CTAB. Figure 7a,d shows that the solvodynamic size of ZIF-8 increases with an increasing amount of 1-mIm, while the ζ-potential decreases except for the one measured with the lowest 1-mIm concentration. 1-mIm as a coordination modulator can compete with 2-mIm, resulting in a faster nucleation rate, thus leading to smaller crystals compared to those in pristine ZIF-8, as we discussed in the previous section. However, on the other hand, the p*K*_a_ value of 1-mIm is lower than 2-mIm, which can prevent the deprotonation of 2-mIm and the intermediate [Zn/2-mIm] clusters, thus slowing the nucleation rate of ZIF-8 and resulting in larger crystal sizes. When we increase the concentration of 1-mIm, the synthesized ZIF-8 in ethanol seems to be influenced greatly by the p*K*_a_ effect, and the sizes were found to be larger than that of the pristine ZIF-8 in ethanol. The decreasing trend of the ζ potential observed in Figure 7a is possibly due to the aggregation of larger particles from higher 1-mIm concentrations. THAM functions as a deprotonation modulator but can also participate in coordination with Zn clusters and chelating with free Zn^2+^. With an increasing amount of THAM, the higher basicity promotes the deprotonation of 2-mIm ligands. We noticed that the overall size trend of THAM-assisted ZIF-8 increased with an increasing amount of THAM added (Figure 6e). We believe that is due to the fact that deprotonated THAM can facilitate cross-linking among preformed ZIF-8 nanocrystals. A single THAM molecule in the ZIF-8 synthesis solution can provide three deprotonated hydroxyl functional groups, which will bind Zn-rich preformed ZIF-8 clusters or nanocrystals through electrostatic interactions, enabling the formation of stable larger crystals. The good affinity between THAM and ZIF-8 clusters promotes the binding between these two; thus, the ζ-potential of THAM-assisted ZIF-8 also increases with an increasing amount of THAM. A similar trend was reported for the Cu nanoparticle (NP)-incorporated graphene quantum dots (Cu-GQDs);^58^ a good binding affinity between GQD and Cu NPs was observed. Therefore, the ζ-potential increased together with the size of the hybrid Cu-GQD with an increasing amount of Cu NPs. In a separate work on studying the effect of THAM on hydroxyapatite NPs in alcohols, a similar trend of increasing ζ-potential with a growth in solvodynamic size was also observed due to the stronger interactions between THAM and the hydroxyapatite NPs through an enhanced hydrogen-bonding.^59^

As for CTAB, the solvodynamic size and ζ-potential of ZIF-8 reach a summit when the CTAB concentration is around 1.0 mM, as shown in Figure 7c. ζ-potential can be influenced by surface adsorption and the thickness of EDL. Surface adsorption alters interfacial charges. CTAB has been confirmed by several previous studies to be adsorbed preferably on the [100] facets of ZIF-8.^32−34^ The CMC of CTAB in methanol is around 1.0 mM.^42^ When the concentration of CTAB in ZIF-8 synthesis solution is below the CMC value, CTAB adsorbs on the surface of ZIF-8 during the crystallization process; thus, a greater amount of CTAB results in a bigger size of ZIF-8 crystals. When the concentration is below the CMC point, this is considered as a lower concentration scenario, where surface adsorption dominates the ζ-potential.^52^ Therefore, a higher concentration of CTAB increases the ζ-potential of formed ZIF-8. On the other hand, when the concentration of CTAB is above the CMC value, micelles and CTAB/alcohol aggregates can be formed;^42^ thus, increasing the amount of CTAB will only create more micelles and aggregates instead of promoting surface adsorption. Hence, it causes the solvodynamic size of ZIF-8 to decrease upon increasing the amount of CTAB. When the concentration is high (greater than CMC in this case), the thickness of EDL dominates the ζ-potential.^52^ With a greater amount of CTAB micelles and CTAB/alcohol aggregates, the thickness of the ZIF-8 EDL will be compressed, thus leading to a lower ζ-potential. As a result, the ζ-potential of the ZIF-8 crystals decreases with an increasing amount of CTAB when its concentration is above the CMC point.

## Conclusions

In summary, we studied the nucleation and crystal growth of ZIF-8 with and without surfactants using time-resolved light dynamic, electrokinetic, and XRD techniques. We reveal that ZIF-8 undergoes a fast nucleation process followed by a fast and then slow crystallization pathway within the first 40 min of synthesis in methanol. 1-mIm, as a coordination modulator, competes with 2-mIm and participates in bonding with Zn clusters, facilitating nucleation and generating smaller crystals compared with those of pristine ZIF-8 in the methanol synthesis solution. But since 1-mIm has a lower p*K*_a_ than that of 2-mIm, the presence of 1-mIm can prevent the deprotonation of 2-mIm, thus the particle size of the resulting 1-mIm-assisted ZIF-8 increases with an increasing amount of 1-mIm in the final ethanol colloids. THAM, as both deprotonation and coordination modulators, increases deprotonation rates of 2-mIm ligands; meanwhile, it functions as a bridging ligand to cross-link ZIF-8 nanocrystals and clusters. CTAB does not affect ZIF-8 nucleation but plays an important role in crystal growth; its effect on the solvodynamic size and zeta potential of ZIF-8 relies on its micelle formation. The novelty of this work includes but is not limited to (1) a deep and systematic understanding of the formation of ZIF-8 using surface techniques; (2) a joint effort of MOF nanoparticle synthesis coupled with X-ray diffraction and electrokinetic studies providing an insight on the relationship between modulators and ZIF-8 nanoparticle growth in colloids; and (3) a focus on the role of interfacial interactions between modulators and MOF crystals toward crystal formation and growth. Our study demonstrates a new approach to study crystal growth of MOFs in situ using a combined DLS and ELS technique. The findings can be applied for using ZIF-8 in heterogeneous catalysis and designing new composite materials in solvents. Moreover, the knowledge learned from this work will advance the development of other hybrid nanocrystal colloid systems.

## Supporting Information Available

Additional AFM and XRD data; DFT calculations; powder XRD simulations; and size and zeta potential raw data (PDF)
