# Supplementary material for: Zeta Potential and Size Analysis of Zeolitic Imidazolate Framework-8 Nanocrystals Prepared by Surfactant-Assisted Synthesis
Source: Langmuir. 2024 Mar 15;40(12):6138–48. doi: 10.1021/acs.langmuir.3c03193 (PMC10976884; doi:10.1021/acs.langmuir.3c03193)
Supplement: Supplementary file 1 — la3c03193_si_001.pdf [file la3c03193_si_001.pdf]

Zeta Potential and Size Analysis of Zeolitic Imidazolate Framework-8 Nanocrystals Prepared by Surfactant-Assisted Synthesis

*Tristan K. Jongert[1], Ian A. Slowinski[1], Benjamin Dao[1]†, Victor H. Cortez[1], Thomas Gredig[2], Nestor D. Plascencia[2], Fangyuan Tian[1]**

1. Department of Chemistry & Biochemistry, California State University Long Beach, Long Beach, CA 90840, USA.

2. Department of Physics & Astronomy, California State University Long Beach, Long Beach, CA 90840, USA.

Supporting Information

Table of Contents

1. Additional atomic force microscopic images…..…………….………………………………*S*-2
2. Additional X-ray diffraction data………..…..……………….……………………………*S-4*
3. Computational details…...……………………….…………….…………………………*S-4*
4. Size and zeta potential distributions……………………………….………………………*S-8*

1. Additional atomic force microscopic (AFM) images

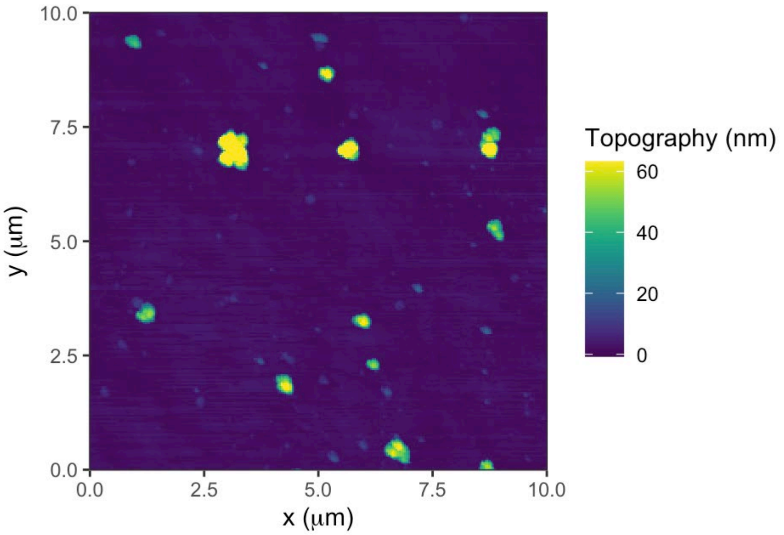

Figure S1. AFM image of pristine ZIF-8 after 10 min synthesis time.

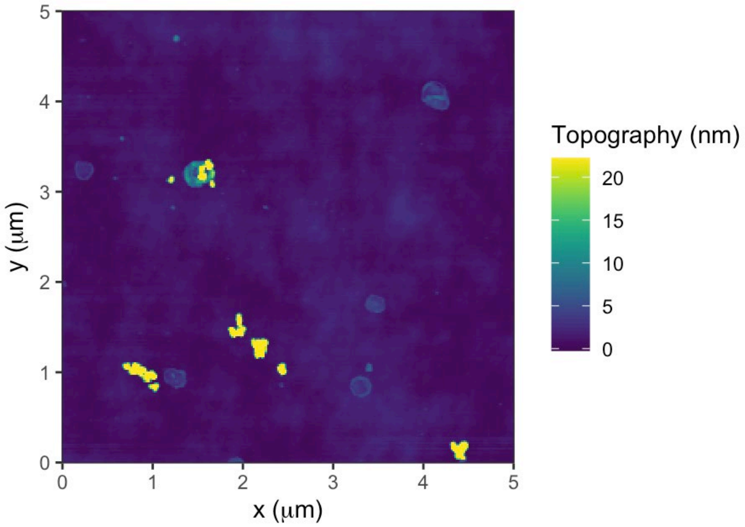

Figure S2. AFM image of ZIF-8 synthesized with additional 2.1 mM of tris(hydroxymethyl)aminomethane (THAM) after 10 min synthesis time.

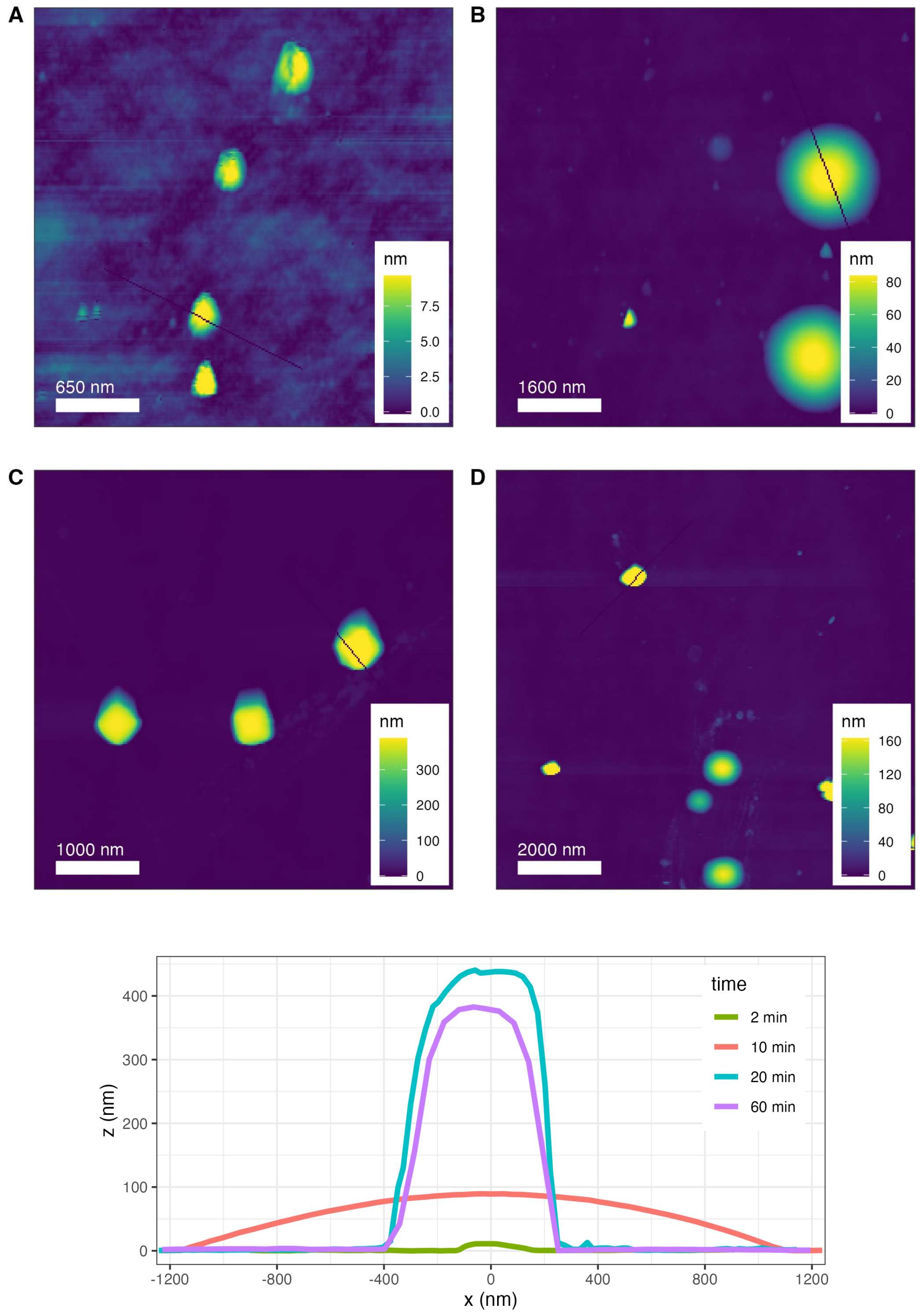

Figure S3. AFM images and cross-line profiles of ZIF-8 synthesized with additional 119 mM of 1-methylimidazole (1-mIm) after 2 min (A), 10 min (B), 20 min (C), and 60 min (D) synthesis time.

2. Additional X-ray diffraction powder data

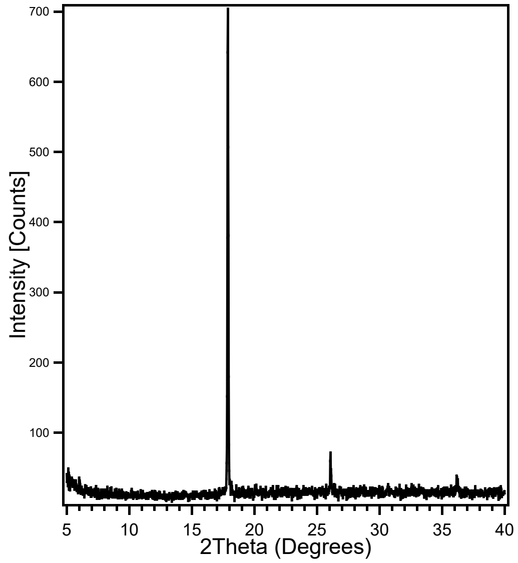

Figure S4. Powder X-ray diffraction pattern of 2-methylimidazole in the 2θ range between 5 and 40º.

3. Computational details.

All calculations were performed with the Gaussian 16 suite of programs (Rev. A.03)[1] using the B3LYP approximation to the exchange-correlated functional[2–4] with LanL2DZ basis set.[5,6] ZIF-8 is represented by a cluster model of Zn[2-mIm]$_4^{2-}$, shown in Fig. S5. For simulations of ZIF-8 interacting with 1-mIm, THAM, and CTAB, one 2-mIm ligand was replaced by a single deprotonated 1-mIm, THAM, or CTAB molecule. All molecular structures were constructed and visualized using GaussView 6 (Rev. 6.0.16). Geometrical optimization of ZIF-8 and surfactants were performed to minimum energy.

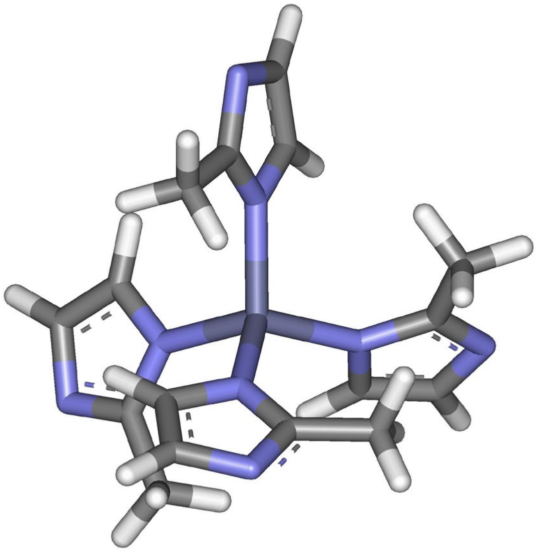

Figure S5. ZIF-8 cluster represented by Zn[2-mIm]$_4^{2-}$. Carbon (grey), nitrogen (blue), zinc (purple), and hydrogen (white) are shown in the optimized structure.

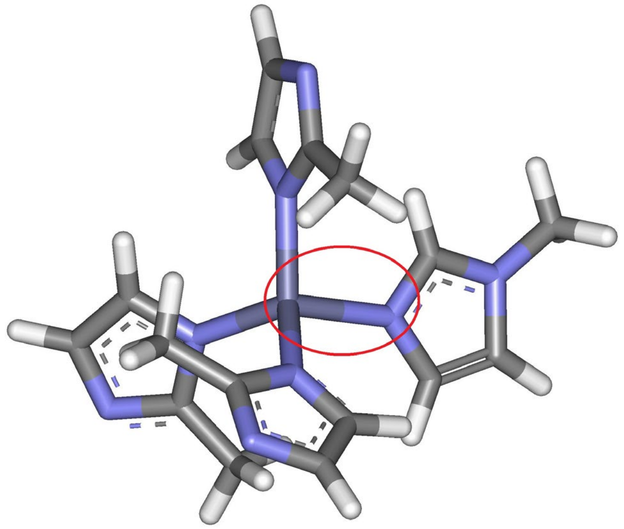

Figure S6. Optimized structure of Zn[2-mIm]$_3$[1-mIm]$^{2-}$. The bond in red circle is between Zn center and 1-mIm ligand. Carbon (grey), nitrogen (blue), zinc (purple), and hydrogen (white) are shown in the optimized structure.

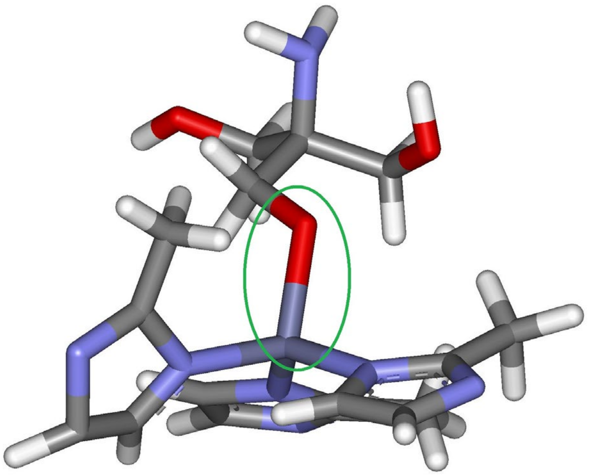

Figure S7. Optimized structure of Zn[2-mIm]$_3$[THAM]$^{3-}$. The bond in green circle is between Zn center and the deprotonated THAM ligand. Carbon (grey), nitrogen (blue), zinc (purple), hydrogen (white), and oxygen (red) are shown in the optimized structure.

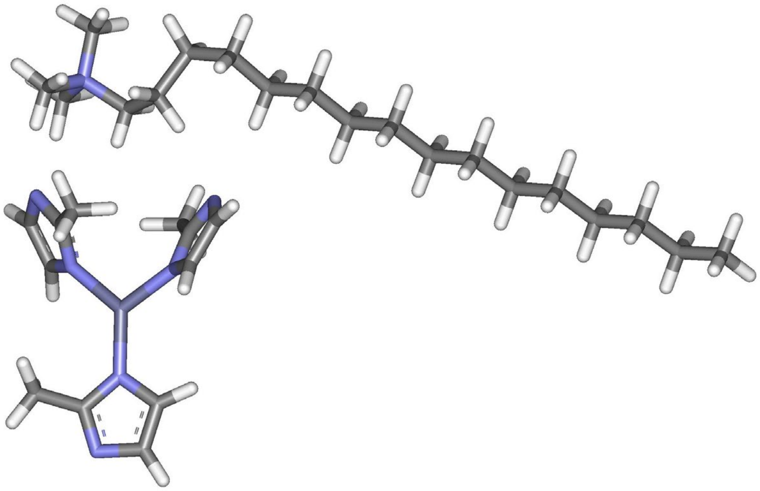

Figure S8. Optimized structure of Zn[2-mIm]$_3$[CTAB]$^-$. Carbon (grey), nitrogen (blue), zinc (purple), and hydrogen (white) are shown in the optimized structure.

Powder XRD pattern of ZIF-8 (SOD, space group I-43m) was simulated using Diamond software with the calculated d-spacing and plane index listed in Table S1.

Table S1. Calculated d-spacings and plane indices of ZIF-8 (Cu Kα radiation, λ=1.5406Å).

2-Theta	d (Å)	hkl
7.334	12.0442	011
10.379	8.5165	002
12.720	6.9537	112
14.698	6.0221	022
16.444	5.3863	013
18.026	4.9170	222
19.484	4.5523	123
20.844	4.2583	004
22.124	4.0147	114
22.124	4.0147	033
23.337	3.8087	024
24.493	3.6314	233
25.600	3.4768	224
26.665	3.3404	134
26.665	3.3404	015
28.683	3.1098	125
29.645	3.0110	044
30.579	2.9211	035
30.579	2.9211	334
31.488	2.8388	244
31.488	2.8388	006
32.375	2.7631	235
32.375	2.7631	116
33.240	2.6932	026
34.085	2.6282	145
34.913	2.5678	226
35.724	2.5114	136
36.519	2.4585	444
37.299	2.4088	055
37.299	2.4088	345
37.299	2.4088	017
38.066	2.3621	046
38.820	2.3179	336
38.820	2.3179	255
38.820	2.3179	127
39.562	2.2761	246

4. Size and zeta-potential distributions.

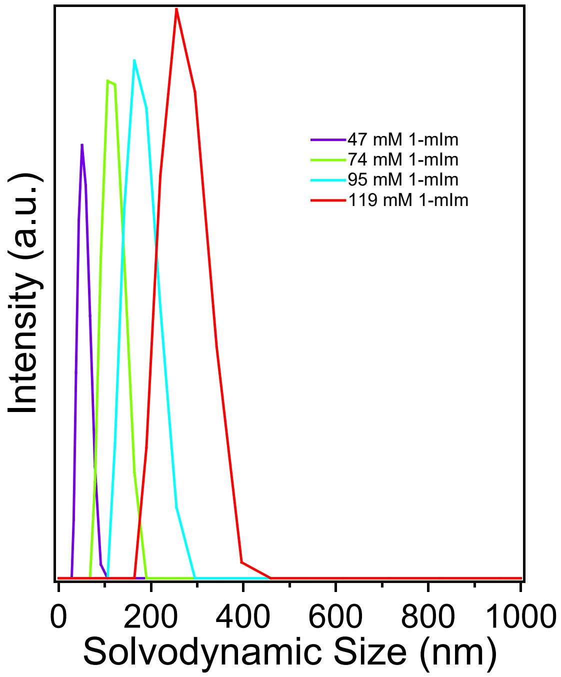

Figure S9. Solvodynamic size distribution of 1-mIm-assisted ZIF-8 in ethanol based on various concentrations of 1-mIm.

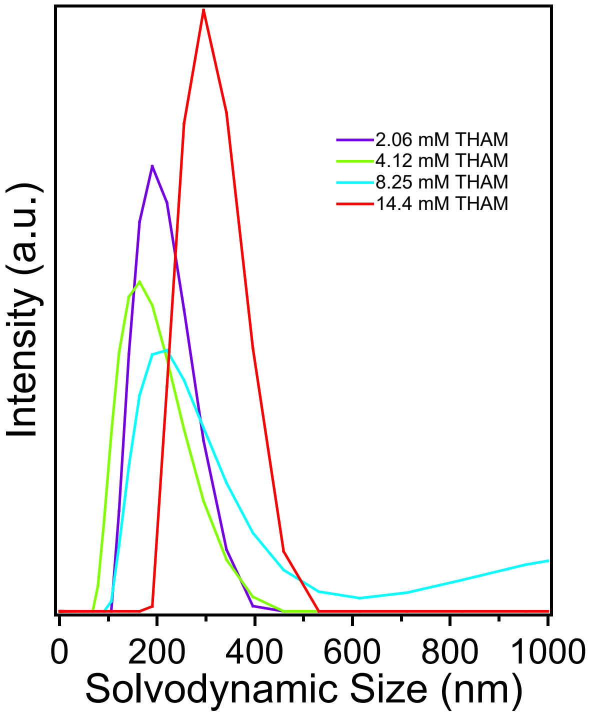

Figure S10. Solvodynamic size distribution of THAM-assisted ZIF-8 in ethanol based on various concentrations of THAM.

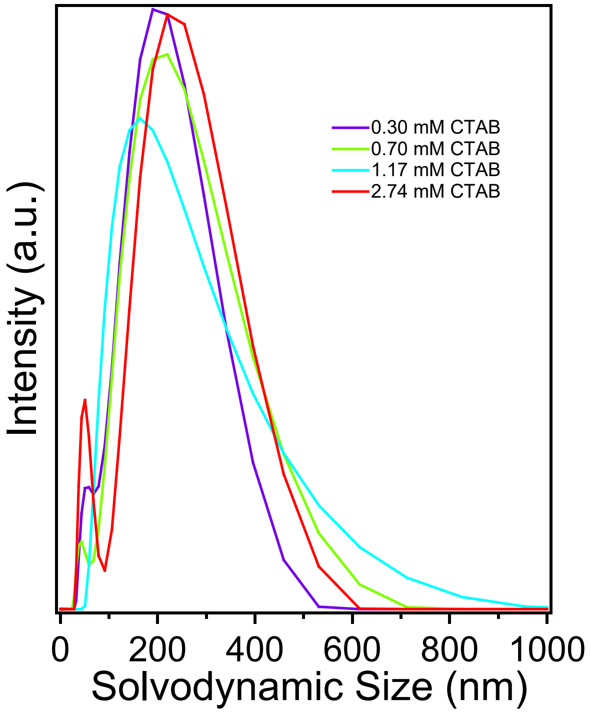

Figure S11. Solvodynamic size distribution of CTAB-assisted ZIF-8 in ethanol based on various concentrations of CTAB.

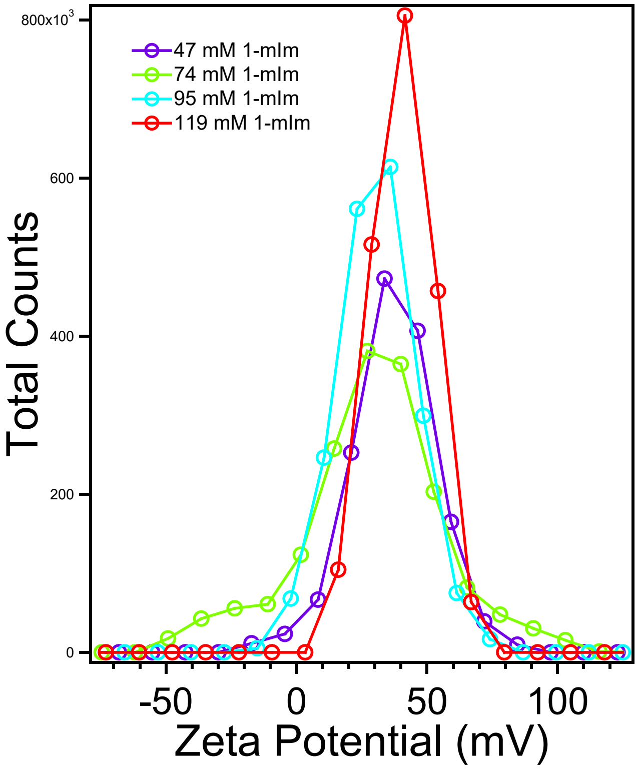

Figure S12. Zeta potential distribution of 1-mIm-assisted ZIF-8 in ethanol based on various concentrations of 1-mIm.

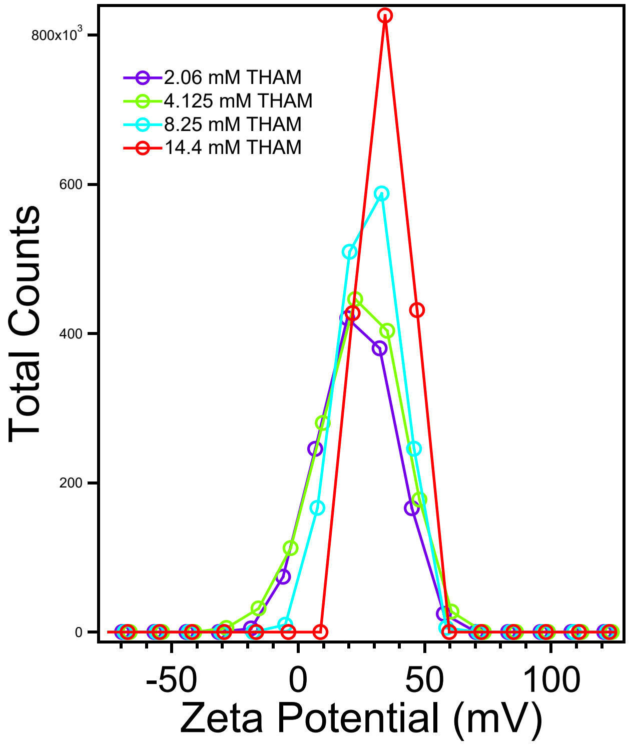

Figure S13. Zeta potential distribution of THAM-assisted ZIF-8 in ethanol based on various concentrations of THAM.

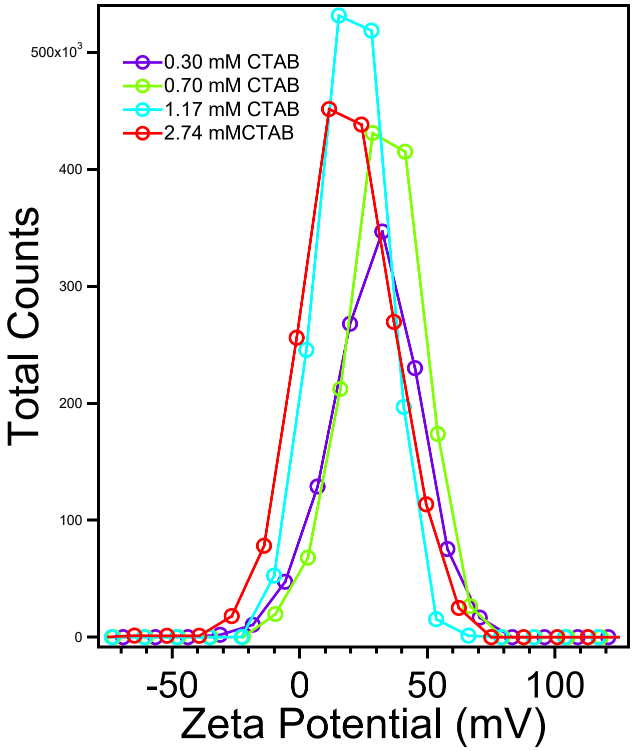

Figure S14. Zeta potential distribution of CTAB-assisted ZIF-8 in ethanol based on various concentrations of CTAB.

References

(S1) Frisch, M. J.; Trucks, G. W.; Schlegel, H. B.; Scuseria, G. E.; Robb, M. A.; Cheeseman, J. R.; Scalmani, G.; Barone, V.; Petersson, G. A.; Nakatsuji, H.; Li, X.; Caricato, M.; Marenich, A. V.; Bloino, J.; Janesko, B. G.; Gomperts, R.; Mennucci, B.; Hratchian, H. P.; Ortiz, J. V.; Izmaylov, A. F.; Sonnenberg, J. L.; Williams-Young, D.; Ding, F.; Lipparini, F.; Egidi, F.; Goings, J.; Peng, B.; Petrone, A.; Henderson, T.; Ranasinghe, D.; Zakrzewski, V. G.; Gao, J.; Rega, N.; Zheng, G.; Liang, W.; Hada, M.; Ehara, M.; Toyota, K.; Fukuda, R.; Hasegawa, J.; Ishida, M.; Nakajima, T.; Honda, Y.; Kitao, O.; Nakai, H.; Vreven, T.; Throssell, K.; Montgomery, J. A., Jr.; Peralta, J. E.; Ogliaro, F.; Bearpark, M. J.; Heyd, J. J.; Brothers, E. N.; Kudin, K. N.; Staroverov, V. N.; Keith, T. A.; Kobayashi, R.; Normand, J.; Raghavachari, K.; Rendell, A. P.; Burant, J. C.; Iyengar, S. S.; Tomasi, J.; Cossi, M.; Millam, J. M.; Klene, M.; Adamo, C.; Cammi, R.; Ochterski, J. W.; Martin, R. L.; Morokuma, K.; Farkas, O.; Foresman, J. B.; Fox, D. J. Gaussian 16. Gaussian, Inc: Wallingford CT 2016.

(S2) Becke, A. D. A New Mixing of Hartree-Fock and Local Density-Functional Theories. *J Chem Phys* **1993**, *98* (2), 1372–1377. https://doi.org/10.1063/1.464304.

(S3) Krishnan, R.; Binkley, J. S.; Seeger, R.; Pople, J. A. Self-Consistent Molecular Orbital Methods. XX. A Basis Set for Correlated Wave Functions. *J Chem Phys* **1980**, *72* (1), 650–654. https://doi.org/10.1063/1.438955.

(S4) Lee, C.; Yang, W.; Parr, R. G. Development of the Colle-Salvetti Correlation-Energy Formula into a Functional of the Electron Density. *Phys Rev B* **1988**, *37* (2), 785–789. https://doi.org/10.1103/PhysRevB.37.785.

(S5) Martin, J. M. L.; Sundermann, A. Correlation Consistent Valence Basis Sets for Use with the Stuttgart–Dresden–Bonn Relativistic Effective Core Potentials: The Atoms Ga–Kr and In–Xe. *J Chem Phys* **2001**, *114* (8), 3408–3420. https://doi.org/10.1063/1.1337864.

(S6) Feller, D.; Peterson, K. A.; de Jong, W. A.; Dixon, D. A. Performance of Coupled Cluster Theory in Thermochemical Calculations of Small Halogenated Compounds. *J Chem Phys* **2003**, *118* (8), 3510–3522. https://doi.org/10.1063/1.1532314.
